# Flavonoids as Promising Antiviral Agents against SARS-CoV-2 Infection: A Mechanistic Review

**DOI:** 10.3390/molecules26133900

**Published:** 2021-06-25

**Authors:** Mohammad Amin Khazeei Tabari, Amin Iranpanah, Roodabeh Bahramsoltani, Roja Rahimi

**Affiliations:** 1 Student Research Committee, Mazandaran University of Medical Sciences, Sari, Iran; aminkhazeeitabari@gmail.com; 2 USERN Office, Mazandaran University of Medical Sciences, Sari, Iran; 3 Pharmaceutical Sciences Research Center, Health Institute, Kermanshah University of Medical Sciences, Kermanshah, Iran; amin.iranpanah75@gmail.com; 4 Student Research Committee, Kermanshah University of Medical Sciences, Kermanshah, Iran; 5 Kermanshah USERN Office, Universal Scientific Education and Research Network (USERN), Kermanshah, Iran; 6 Department of Traditional Pharmacy, School of Persian Medicine, Tehran University of Medical Sciences, Tehran P.O. Box 1417653761, Iran; Roodabeh.b.s.88@hotmail.co.uk; 7 Research Center for Clinical Virology, Tehran University of Medical Sciences, Tehran, Iran; 8 PhytoPharmacology Interest Group (PPIG), Universal Scientific Education and Research Network (USERN), Tehran, Iran

**Keywords:** inflammation, lung, oxidative damage, antiviral, polyphenol

## Abstract

A newly diagnosed coronavirus in 2019 (COVID-19) has affected all human activities since its discovery. Flavonoids commonly found in the human diet have attracted a lot of attention due to their remarkable biological activities. This paper provides a comprehensive review of the benefits of flavonoids in COVID-19 disease. Previously-reported effects of flavonoids on five RNA viruses with similar clinical manifestations and/or pharmacological treatments, including influenza, human immunodeficiency virus (HIV), severe acute respiratory syndrome (SARS), Middle East respiratory syndrome (MERS), and Ebola, were considered. Flavonoids act via direct antiviral properties, where they inhibit different stages of the virus infective cycle and indirect effects when they modulate host responses to viral infection and subsequent complications. Flavonoids have shown antiviral activity via inhibition of viral protease, RNA polymerase, and mRNA, virus replication, and infectivity. The compounds were also effective for the regulation of interferons, pro-inflammatory cytokines, and sub-cellular inflammatory pathways such as nuclear factor-κB and Jun N-terminal kinases. Baicalin, quercetin and its derivatives, hesperidin, and catechins are the most studied flavonoids in this regard. In conclusion, dietary flavonoids are promising treatment options against COVID-19 infection; however, future investigations are recommended to assess the antiviral properties of these compounds on this disease.

## 1. Introduction

By the end of 2019, an unusual pneumonia was reported from China which was further diagnosed as a novel coronavirus (CoV) causing severe acute respiratory syndrome (SARS) and was called COVID-19 [1,2]. Later, the virus (SARS-CoV-2) spread to other countries and was declared a pandemic by WHO on 11 March 2020 [3]. The virus is transmitted mostly by respiratory droplets, and the severity ranges from mild to severe lethal symptoms. The asymptomatic cases in the incubation period are thought to be an important source of contagion [4]. In most cases, mild symptoms take 1–2 weeks to resolve, whereas severe cases can lead to death [5].

SARS-CoV-2 affects the respiratory system, causing fever and dry cough [4]; however, the virus can cause organ failure, mainly in the heart and kidneys, as well as causing cytokine storms, which further increase mortality. The viral life cycle of SARS-CoV-2 includes attachment, penetration, biosynthesis, maturation, and release. After the attachment, viral RNA enters the cell nucleus for replication, and viral mRNA starts generating viral structural proteins, including the spike (S), membrane (M), envelope (E), and nucleocapsid (N) proteins [6]. The angiotensin-converting enzyme 2 (ACE2) receptor, being highly expressed in the lungs, has also been shown to act as a co-receptor for SARS-CoV [7].

SARS, MERS, and SARS-CoV-2 are all RNA βCoVs. SARS-CoV-2 genome is 88% identical to bat-derived severe acute respiratory syndromes (SARS)-like CoVs, 79% similar to SARS-CoV, and 50% similar to MERS-CoV [8,9]. SARS-CoV-2 proteins are 90%–100% homologous to SARS-CoV; though, orf10 and orf8 are different between SARS-CoV-2 and SARS-CoV. Orf8 is an accessory protein for βCoVs. It forms a six strand alpha helix protein that enhances the virus’s ability to spread. SARS-CoV-2 orf1a/b, spike, envelope, membrane, and nucleoprotein are also closely related to those of SARS-CoV [10].

Since there is no specific antiviral agent against SARS-CoV-2, currently available antiviral drugs are considered for the treatment of COVID-19. Remdesivir is a new antiviral drug specifically introduced for the Ebola virus in 2015. It has an inhibitory effect on viral RNA polymerase and has recently been used in some trials for COVID-19 treatment [11]. Favipiravir has an inhibitory effect on influenza and the Ebola virus with the same mechanism and is also assessed in SARS-CoV-2 [12,13,14]. Lopinavir is a viral protease inhibitor and was firstly developed for HIV treatment. In vitro studies showed an inhibitory effect of lopinavir in CoV-infected cells [15,16]; however, systematic reviews failed to show any beneficial effect against SARS-CoV-2 [17].

Medicinal plants have been a reliable source of natural drugs, including antiviral agents, since ancient times. Traditional medicine and ethnopharmacological studies of different countries all over the world have always opened new ways for drug discovery [18,19,20,21,22]. Oseltamivir which is a conventional antiviral agent, is a derivative of shikimic acid, a secondary metabolite of star anise (*Illicium verum* Hook.f.). In the case of SARS-CoV-2, in silico studies have revealed the possible antiviral properties of herbal ingredients [23,24,25]. Flavonoids are a large class of phytochemicals commonly found in several foods and vegetables in the human diet with numerous valuable pharmacological activities, including antiviral properties. It is demonstrated that these compounds can inhibit viral pathogenesis targeting essential stages of the viral life cycle [26]. Quercetin, catechins, kaempferol, and baicalein are examples of the most important flavonoids exhibiting antiviral properties [18,27,28]. This study aims to discuss the available antiviral evidence of flavonoids as a possible treatment against SARS-CoV-2 considering the previously-reported effects of these compounds on five RNA viruses with similar clinical manifestations and/or pharmacological treatments, including influenza, human immunodeficiency virus (HIV), severe acute respiratory syndrome (SARS), Middle East respiratory syndrome (MERS), and Ebola.

## 2. Results

Antiviral activity of flavonoids can be categorized into direct antiviral effects where the virus is directly affected by the flavonoid, and indirect effects where the flavonoid improves host defense mechanisms against viral infection. Here, the underlying antiviral mechanisms of flavonoids are discussed with reference to the most important flavonoids demonstrating these mechanisms.

### 2.1. Direct Antiviral Mechanisms

#### 2.1.1. Inhibition of Viral Protease

Viral proteases are used for cleaving the viral polyprotein precursors at certain places to release functional proteins. Specific viral proteases have also been shown to cleave host cell proteins, including translation initiation factors (eIF4 and eIF3d) in HIV, to prevent host protein translation [29,30]. Coronaviruses generate three types of viral proteases, including 3-chymotrypsin-like cysteine (3CLpro), papain-like protease (PLpro), and main protease (M pro) [31]. 3CLpro is important for the SARS-CoV life cycle, PLpro plays a role in SARS-CoV-2 replication, and Mpro is responsible for the maturation of functional proteins in SARS-CoV-2 [32,33]. As a result, these molecules are suitable drug targets in antiviral research and drug discovery [34].

Kaempferol which is an abundant flavonoid in several foods decreased CPE in Vero E6 cells infected with clinical isolates of SARS-CoV-2 with around 88% of inhibition at 125 μM concentration. A coupled in silico investigation suggests inhibition of SARS-CoV-2 3CLpro enzyme to be the main mechanism of action [35]. The addition of kaempferol to 3CLpro and PLpro of SARS-CoV and MERS-CoV expressed in *E. coli* caused antiviral effects via inhibition of these enzymes [36]. Park and coworkers (2017) showed that the presence of the hydroxyl group in kaempferol causes a more potent antiviral activity through inhibition of 3CLpro and PLpro [37]. Epigallocatechin gallate (EGCG) is a flavonoid found in tea (*Camellia sinensis* L.) with antifungal, antibacterial, and antiviral properties [38,39]. Studies indicated that EGCG inhibits reverse transcriptase (RT) activity, protease activity, p24, viral entry, and viral production in THP-1 and H9 cells infected with HIV-1, and liposome modification of EGCG amplified its inhibitory effects. Cell-free studies also showed significant downregulation in protease kinetics after treatment with EGCG. The galloyl group in EGCG is considered to be responsible for its antibacterial and antiviral activities [40]. Isoliquiritigenin, a chalcone, can be used as a therapeutic agent in bacterial and viral infections [41]. This compound has shown an inhibitory effect on SARS-CoV, and MERS-CoV 3CLpro and PLpro expressed in *E. coli*. The presence of a prenyl functional group on the resorcinol ring allows the formation of hydrophobic interactions with proteases [37]. Theaflavins are polyphenols found in various kinds of tea [42]. Experiments on SARS-CoV recombinant protease showed a significant reduction in 3CLpro activity after treatment with theaflavin-3,3′-digallate with an IC50 value of 9.5 μM. The gallate group attached to the 3′ position in some theaflavins might be essential for interaction with the 3CL proactive site [43]. Prenylisoflavonoids extracted from *Erythrina senegalensis* DC. were used to evaluate anti-protease activity against recombinant HIV-1 protease. The results showed that the compounds could inhibit HIV-1 recombinant protease in vitro with 0.5 to 30 μM IC50 values. Hydroxy and pernyl groups might be responsible for the inhibition of HIV protease [44]. Quercetin and quercetin-β-galactoside downregulated PLpro, 3CLpro, deubiquitination, and DeISGylation activity in SARS-CoV, MERS-CoV, and HIV-1. The position of hydroxyl groups might be effective in anti-protease activity. Quercetin with five hydroxyl groups at the 3,5,7,3′ and 4′ positions exhibited a strong inhibitory effect on viral proteases; while the presence of glycosyl group at position 3,7,4′ reduced the inhibitory effect [37,45].

#### 2.1.2. Inhibition of Viral RNA Polymerase and Viral mRNA

RNA-dependent RNA polymerase (RdRp) is an important enzyme catalyzing the replication of RNA from an RNA sequence [46]. This enzyme is encoded in all RNA viruses, as well as some eukaryotes [47]. Viruses are obligate intracellular parasites, i.e., they cannot independently survive out of cells. They must use cellular translational equipment to translate mRNAs for protein production, which is required for replication. Thus, any interference with mRNA translation would inhibit viral replication, spread, and evolution [48].

A recent investigation by Zandi et al. revealed the in vitro antiviral effect of baicalin and baicalein against SARS-CoV-2 infection in Vero CCL-81 cell line through inhibition of RdRp, with a higher potency by baicalein. Further in silico evaluations showed these two compounds to have a higher affinity to RdRp in comparison to remdesivir. The attachment site of baicalin and baicalein also seems to be different from that of remdesivir; thus, these flavonoids can be used as an adjuvant treatment along with remdesivir [49]. The effects of quercetin-7-O-glucoside (Q7G) were assessed in an in vitro study on MDCK cells infected with influenza viruses A and B in comparison to the standard antiviral agent oseltamivir. Oseltamivir was used as a control drug and showed moderate antiviral activity with IC50 values 25.4 to 42.2 μg/mL; while Q7G inhibited influenza A and B virus with IC50 value 3.10 μg/mL to 8.19 μg/mL. Oseltamivir also showed a weaker activity against influenza B than influenza A; whereas Q7G demonstrated strong activity against all influenza viruses. Additionally, quantitative PCR assays reported a higher decrease in viral RNA synthesis after Q7G treatment compared with oseltamivir, indicating the inhibitory effect of Q7G on viral RNA polymerase. Molecular docking analysis revealed this interaction to be due to the attachment of Q7G to the PB2 subunit of viral RNA polymerase [50].

Oroxylin A (OA) is a flavonoid found in *Oroxylum indicum* (L.) Kurz. It has been shown that OA can inhibit several influenza A strains in MDCK cells in a dose-dependent manner. Oral treatment of mice infected with influenza virus H1N1 with OA also decreased virus-induced death, bodyweight loss, and lung injury, with a survival rate of 60.0% at 100 mg/kg daily dose. Antiviral effects of OA were reported to be due to downregulation of H1N1 matrix 1 (M1) mRNA transcription and protein synthesis (Jin et al. 2018). The M1 protein is a protein within the viral envelope that binds to the viral RNA and can mediate encapsidation of RNA nucleoprotein cores into the membrane envelope [51]. Although OA could inhibit protein synthesis, it could not block viral entry to host cell or nucleoprotein (NP) entrance to host cell nucleus [52]. Baicalin and biochanin A could inhibit influenza H5N1 infection in A549 cells with the IC50 values of 18.79 and 8.92 μM, respectively. This effect was mediated by suppressing nuclear viral ribonucleoprotein (RNP) export [53]. Other studies have also shown that baicalin can downregulate influenza M1 protein expression [54,55].

Host cdc2-like kinase 1 (CLK1) has a key role in the splicing of the H1N1 influenza virus M2 gene and is an important anti-influenza target. M2 is a proton channel in the viral envelope of the influenza A virus [56,57]. It was demonstrated that gallocatechin-7-gallate isolated from *Pithecellobium clypearia* is an inhibitor of host cdc2-like kinase 1 (CLK1), an anti-influenza target due to its role in viral M2 mRNA alternative splicing. Investigations on the effect of gallocatechin-7-gallate at the daily dose of 30 mg/kg on ICR mice infected with H1N1 virus showed a significantly higher survival up to 8 days. It also inhibited virus-induced acute lung injury and weight loss. Additionally, assessments on H1N1-infected A549 cells demonstrated a significant downregulation of viral NP and M2 mRNAs. Moreover, the phosphorylation of splicing factors SF2/ASF and SC35, key factors for virus M2 gene alternative splicing, was significantly decreased after treatment with gallocatechin-7-gallate [58]. Cirsimaritin (CST), a flavonoid from *Artemisia scoparia* Waldst. and Kitam was assessed regarding its in vitro antiviral effects on MDCK and THP-1 cells infected with three influenza virus strains which showed IC50 values ranging from 5.8 to 11.1 μg/mL, compared with 3.4 to 8.9 μg/mL for ribavirin. Data demonstrated that CST could effectively reduce influenza M2 and protein expression in a dose-dependent manner so that the potency of CST at 20 μg/mL was higher than 10 μM of the standard antiviral ribavirin [59]. Luteolin is another flavonoid with an inhibitory effect on M2 mRNA expression. In MDCK cells infected with different influenza strains, 15 μM of luteolin was more effective than 10 μM of oseltamivir in both H1N1 and H2N3 infected cells. Luteolin also downregulated influenza virus coat protein I (COPI) expression, mediating virus entry and endocytic pathway, in infected cells [60]. Santin is a flavonoid extracted from *Artemisia rupestris* L., which was also suggested to have anti-influenza virus effects through suppression of M2 mRNA expression in a dose-dependent manner [61].

It was indicated that quercetin could be a probable therapeutic agent against influenza infection at the early stages of infection so that it can be used for influenza virus prophylaxis. Investigations on the effects of quercetin on MDCK and A549 cells infected with influenza virus A strains revealed that it could inhibit viral NP mRNA in a dose-dependent manner, with the highest activity at 50 μM concentration [62].

Research has shown that tricin (4′,5,7-trihydroxy-3′,5′-dimethoxyflavone) exhibits antiviral activities against influenza A and B strains. RT-PCR tests indicated that tricin could suppress M protein mRNA synthesis in MDCK cells infected with influenza virus; with no significant effects on neuraminidase and hemagglutinin biosynthesis. The 50% effective concentration of tricin, which could inhibit viral mRNA synthesis, was 3.4–10 µM for influenza A virus strains and 4.9 µM for influenza B virus. In mice infected with influenza virus, tricin with a dose of 20 µg/kg ameliorated body weight loss and survival time [63].

#### 2.1.3. Viral Entry, Replication, and Infectivity

RNA viruses encode proteins utilizing the host cellular machinery for their life cycle. Understanding these host cell necessities not only informs us of the molecular pathways used by the virus, but also presents additional targets for drug development [64].

An in vitro study assessed the effect of quercetin and isorhamnetin on SARS-CoV-2 entry to ACE2h cells. ACE2 expressed on lung cells is a co-receptor of viral spike protein and, thus, is a main target of antiviral agents against SARS-CoV-2. It was observed that these two flavonoids have a high binding affinity to ACE2 and subsequently decrease viral entry via the inhibition of spike protein attachment to this receptor [65]. Another study assessed the effect of baicalein on SARS-CoV-2 infection in Vero E6 cells and hACE2 transgenic mice. A significant reduction was observed in in vitro and in vivo viral replication, as well as body weight loss and lung injury of animals [66]. Dihydroxy-6′-methoxy-3′,5′-dimethylchalcone and myricetin-3′,5′-dimethyl ether 3-*O*-β-d-galactopyranoside are flavonoids derived from *Cleistocalyx operculatus* (Roxb.) Merr. and L.M. Perry. Cytopathic effect (CPE) reduction assay showed that these flavonoids inhibit viral replication of influenza virus H1N1 in MDCK cells. Structure-activity relationship (SAR) studies indicated that OH groups at C-7 and C-4, a double bond between C-2 and C-3, and especially a carbonyl group at the C-4 position, are critical functional groups that significantly improve the antiviral properties of flavonoids [67]. 3-deoxysappanchalcone (3DSC) isolated from *Caesalpinia sappan* L. could inhibit influenza virus replication in high concentrations via inhibition of viral NP expression in MDCK cells infected with the H1N1 virus. At an equal concentration (30 µM), both ribavirin and 3DSC showed significant inhibition of NP expression, though ribavirin had a stronger effect [68]. Studies have demonstrated that biochanin A and baicalein inhibited caspase-3 activation, an enzyme involved in viral replication [53,69]. These compounds could also inhibit the nuclear export of viral RNP complexes, which is critical in viral replication [53]. Biochanin A showed an inhibitory effect against ½ mitogen-activated p38 and NF-κB, which were shown to be involved in viral replication. NF κB and p38 are activated due to oxidative stress and are known to affect influenza A virus replication and pathology [53,70]. Investigations on cell cultures of MDCK cells and A549 cells infected with influenza virus showed that baicalein could inhibit viral replication at 20–80 µg/mL concentrations. Interestingly, baicalin showed similar antiviral activity to ribavirin and oseltamivir at concentrations of 40 µg/mL and 60 µg/mL, respectively. Baicalin also inhibited viral replication in the lungs of mice in vivo [71]. CST was shown to downregulate NF-κB protein and NF-κB phosphorylation in the nucleus [59]. It is already known that NF-κB has an important role in inflammation, oxidative stress, and host immunity suppression [72]. The downregulation of NF-κB also inhibits replication in various types of viruses, including influenza virus [73].

In Vero E6 cells infected with SARS-CoV-2, naringenin could inhibit CPE in a time- and concentration-dependent manner. This effect was mediated through inhibition of endo-lysosomal Two-Pore Channels (TPCs), a pathway involves in infectivity of SARS-CoV-2, Ebola, and MERS via facilitating viral entry [74]. EGCG has shown a dose-dependent inhibitory effect (25, 50 µM) on HIV replication in T-cells; however, inhibition of viral replication was not directly affected by RT inhibition. Fassina et al. analyzed the p24 enzyme, which is involved in packaging viral particles. The results showed a downregulation of p24 concentration and RT activity in HIV-infected T lymphoblasts. Based on the following results, it was noted that EGCG inhibited viral replication through the downregulation of viral infection. There is yet no certainty about the exact effect of EGCG on viral infection [75]. gp120 signaling is commonly associated with increased HIV-1 replication in previously infected cells [75]. Studies show the inhibitory effect of genistein on gp120 and, subsequently, HIV-1 viral replication. There was no change in viral replication after administration of genistein with a concentration of 1–2.5 µg/mL, but in a range of 5–10 µg/mL, genistein could suppress viral replication [76]. Herbacitrin is a flavonoid derived from *Drosera peltata* Thunb. and was previously known as an antiviral agent. It was shown that herbacitrin inhibits both RT and integrase in HIV-1 infected MT-4 and MT-2 cell cultures, resulting in viral replication blockade at different stages. At a concentration of 21.5 µg/M, herbacitrin could suppress RT activity, while it could inhibit integrase at a lower concentration, 2.15 µM [77]. Scutellarin purified from *Erigeron breviscapus* is a flavonoid with anti-HIV-1 activity. This flavonoid inhibited HIV-1 RT activity and cell fusion as major participants of viral replication [78]. Hesperidin and linarin are flavonoids with rutinose at the A ring and methoxy (-OCH3) substitution at the B ring. Isoquercetin has been shown to inhibit influenza A and B virus replication in infected MDCK cells. The combination of isoquercetin with amantadine also showed a synergistic effect on viral replication in MDCK cells infected with influenza A virus only in low doses (0.5 µM for isoquercetin and 1 µM for amantadine). Virus titer values after administration of isoquercetin and amantadine were about 7.5; while increasing isoquercetin and amantadine concentrations lowered the synergistic effect on virus titers to the value of 5 [79]. Q3R derived from *Houttuynia cordata* exerts anti-influenza virus effects. The effects of Q3R on MDCK cells infected with influenza virus A were compared to oseltamivir. Pulmonary lesions and edema were inhibited by Q3R more than oseltamivir. Q3R also had a higher efficacy compared to oseltamivir. The inhibitory effect of Q3R on influenza virus replication was indirect and through interaction with viral particles. Oseltamivir demonstrated moderate antiviral activity, about 58% against influenza A virus, and weak antiviral activity less than 49% with doses under 10 µg/mL; while Q3R showed 86% viral inhibition at 100 µg/mL and 66% inhibition in 10 µg/mL concentrations [80]. Quercetin 3-β-O-D-Glucoside (Q3G) was shown to prevent Ebola virus replication in vitro. Prophylactic administration of Q3G 30 min before the infection showed significant prevention of the Ebola virus. Q3G could also inhibit viral entry at the early stages. So Q3G could be an effective flavonoid for Ebola virus prophylaxis [81].

### 2.2. Indirect Antiviral Effects

#### 2.2.1. Effect on Interferons

Interferons (IFNs) comprise a group of proteins produced by several immune cells in response to many pathogens like viruses, parasites, bacteria, and tumor cells. There are three major classes of IFNs, including type I or acid-stable interferons (IFN-α subtypes, IFN-β, IFN-κ, IFN-ɛ, IFN-ω, and IFN-τ), type II (IFN-γ), and type III IFNs that known as IFN-λ [82,83,84]. They show a wide range of biological activities like activation of the innate immune response, increasing the expression of major histocompatibility complex (MHC) molecules, suppressing angiogenesis. Their most important role is to interfere with viral infections [84,85].

In the early phases of viral infection, IFNs activate the innate immune system. Recent studies have reported a decrease in the type I and type II IFN induction and signaling in COVID-19 patients [86]. These types of IFN have demonstrated antiviral effects by decreasing neutrophils immigration to the inflammation site, increasing antigen presentation, suppression of mononuclear macrophage-mediated pro-inflammation, and activating the acquired immunity for the progression of antigen-specific B and T cell responses [86,87,88,89,90]. Thus, IFNs usage at the early phase of the disease could decrease symptoms of the COVID-19 by reducing viral replication. Researchers also reported that IFN-γ levels could increase in COVID-19 patients with ARDS. The rapid rise in IFNs levels could invite pro-inflammatory cytokines into the alveolar tissue and resulting in pulmonary inflammation and lung injury [90,91]. Therefore, it seems that either upregulation or dysregulation of IFNs and other pro-inflammatory cytokines responses or both could exert a significant role in the progression and pathological features of SARS-CoV-2.

Li et al. investigated the anti-influenza effects of baicalin, a glycosyloxyflavone that is the 7-O-glucuronide of baicalein, in the in vitro and in vivo model of influenza A virus infection. TNF receptor-associated factor 6 (TRAF6) is an effective mediator in the IFN production signaling pathway. Overexpression of TRAF6 leads to increased production of type I IFN [54]. MicroRNAs (miRNAs) are small molecules that control gene regulation post-transcriptionally [92]. miR-146a has been shown to have a regulatory role in inflammation [93]. miR-146a could enhance the replication of H1N1 and H3N2 through the downregulation of TRAF6. Baicalin (20 μg/mL) indicated a significant reduction in the miR-146a expression, viral NP, M1 protein levels, viral titer, and also increased mice survival rate [54]. In another study, Nayak and colleagues represented anti-influenza virus (H1N1-pdm09) activity of baicalin through regulating viral protein NS1, resulting in up-regulation of interferon regulatory factor 3 (IRF-3), IFN-γ, and IFN-β. This IFN up-regulation decreased viral replication that could reduce viral transcripts and pro-inflammatory cytokines expression, including IL-8 and TNF-α [55].

Ding et al. designed a study to investigate the effects of hesperidin, a flavanone glycoside, in the influenza A virus (H1N1)-induced lung injury in male rats. The results showed that hesperidin attenuated lung injury via decreasing pro-inflammatory cytokine production, including IFN-α, TNF-α, and IL-6, through suppressing MAPK signaling pathways. Hesperidin also decreased IFN-α in the H1N1 infected pulmonary microvascular endothelial cells [94]. In another study by Kim et al. isoquercetin effectively attenuated lung injury induced by the H1N1 virus in mice via reducing IFN-γ, iNOS, RANTES, virus titers, viral bronchitis, and bronchiolitis [79].

Oroxylin A (OA) from *Oroxylum indicum* (L.) Kurz prevented the lung injury induced by influenza A H1N1 virus in mice via up-regulation of IFN-β and IFN-γ [52]. Wogonin, another flavonoid isolated from *Scutellaria baicalensis* Georgi, exhibited a significant anti-influenza activity by regulation of AMPK pathways. Wogonin also increased the regulation of IFN-β, IFN-λ1, and IFN downstream molecules, including myxovirus resistance gene A (MxA) and 2-5′ oligoadenylate synthetase (OAS), in MDCK and A549 infected cells [95].

#### 2.2.2. Effect on Pro-Inflammatory Cytokines (TNF, IL, and MCP)

CoVs contain some open reading frames which encode a few accessory proteins. These accessory proteins have been shown to modulate inflammatory pathways such as IFN signaling and pro-inflammatory cytokines [96]. It has been elucidated that the prognosis of COVID-19 could be worsened by the secretion of pro-inflammatory cytokines, including interleukins, IFNγ, and TNF-α [97]. Blanco-Mello et al. indicated that inappropriate immune response might help virus replication and complications due to severe types of COVID-19 [98]. Ruan et al. also showed that an elevation in inflammatory cytokines such as IL-6 is associated with ARDS, respiratory failure, and adverse clinical outcomes [99]. Respiratory failure caused by lung damage is a result of the overproduction of pro-inflammatory cytokines after the infiltration of immune cells into the lung [100]. Cytokine storm is a systemic inflammatory response associated with a broad range of factors like infections and certain drugs. Several studies showed a significant connection between the cytokine storm, severe inflammation, and multiple organ failure in COVID-19 patients [101,102,103]. SARS-CoV-2 virus recognition with innate and adaptive immune systems could result in the activation and production of inflammatory cytokines. According to recent studies, plasma levels of pro-inflammatory cytokines are enhanced in COVID-19 patients. These inflammatory cytokines like TNF-α, IL 6, IL 2, IL-1β, IL 7, IL 10, and IL-18, as well as monocyte chemoattractant protein-1 (MCP-1), have pivotal roles in pathological progression and severity of COVID-19 through an increase in viral load, pneumonia, lung damage, neurological disorders, and mortality [97,101].

These events could lead to multi-organ failure and lung injury as the main complication of SARS-CoV-2; therefore, modulation of pro-inflammatory cytokines can be considered as a reasonable treatment goal in COVID-19. In addition, significant anti-inflammatory effects of flavonoids have been demonstrated in many studies; thus, they may be promising compounds in combating inflammation-related complications of COVID-19 [97].

Yang et al. proved the protective effect 3-deoxysappanchalcone (30 µM) on in vitro influenza H1N1 virus-induced inflammation and apoptosis by decreasing IL-1β and IL-6 levels [68]. Baicalein, a flavone, and biochanin A, an O-methylated isoflavone, reduced pro-inflammatory cytokine expression in A549 cells and primary human monocyte-derived macrophages (MDM) infected with influenza H5N1 virus strains, that could prevent inflammatory pathway activation and tissue damages [53]. In influenza A-infected A549 and MDCK cells, baicalin, the glycosylated form of baicalein (baicalein-7-glucuronide), could increase IFN levels, resulting in a reduction of pro-inflammatory cytokines production. Thus, IL-8 and TNF-α were significantly lower in baicalin-treated cells compared with the untreated control cells [55].

An in vitro study has shown that 2.5, 5, and 10 μg/mL concentrations of CST, a dimethoxyflavone, has a significant effect on the attenuation of NF-κB signal transduction pathway in THP-1 cells infected with influenza A (H1N1) virus. Following NF-κB inhibition, the production of pro-inflammatory cytokines including IL-1β, IL-8, IL-10, and TNF-α, as well as the inflammation-related protein COX-2, were suppressed by CST in a dose-dependent manner [59]. In an in vitro study by Yonekawa et al. on the antiviral properties of hesperidin and linarin, these flavonoid glycosides inhibited R5-HIV-1-NL(AD8) viral replication in CD4+ NKT cells by increasing the production of anti-inflammatory cytokines including IL-2, IL-5, and IL-13. It was observed that the stimulatory effect of these two flavonoids are critically dependent on the sugar moiety as the aglycones (hesperetin and acacetin) failed to show such activity. Furthermore, methoxy (-OCH3) substitution at the B ring is essential for the stimulatory activity of hesperidin and linarin on CD4+ NKT cells. They could also induce RANTES, MIP-1α, and MIP-1β secretion from Vδ1+ expressing T cell receptors which subsequently suppressed viral replication in CD4+ NKT cells [104]. Kang et al. reported anti-influenza effects of purified flavonoids from *Pithecellobium clypearia* Benth on the in vitro model of influenza A virus infection. These Purified flavonoids suppressed the production of IL-6 and MCP-1 in H1N1-infected human A549 lung cells [105]. Mehrbod et al. investigated the anti-inflammatory effect of quercetin-3-O-α-L-rhamnopyranoside (Q3R), a glycosylated flavone, on MDCK cells infected with influenza H1N1 virus. Q3R at 150 μg/mL concentration significantly decreased virus titer and increased IL-27 production, which could further elevate IL-10 secretion by CD4+ T cells and enhance their antiviral activity. On the other hand, Q3R suppressed TNF-α production as one of the important inflammatory mediators causing fever and triggering NF-κB pro-inflammatory pathway, further worsening the condition of patients [106]. The trimethoxyflavone santin has demonstrated anti-influenza activity in THP-1 and MDCK cells in a 60 µM concentration. Influenza A (H3N2) virus induces pro-inflammatory cytokine production in THP-1 cells that results in lung inflammation and injury [61]. Anti-inflammatory cytokines might also be altered during influenza virus infection. IL-10 is an anti-inflammatory cytokine that can be induced by influenza virus. IL-10 inhibits invariant natural killer T cells by downregulating the production of IL-12 by pulmonary monocyte-derived dendritic cells [107]. The levels of IL-6, IL-8, IL-10, IL-1β, and TNF-α were significantly decreased in the santin-treated group through downregulation of MAPKs and NF-κB signaling pathways [61].

In addition to the above, gallocatechin-7-gallate, genistein and theaflavins are other flavonoids with modulating effects on the production of pro-inflammatory cytokines [58,76,108]; thus, these molecules seem to have a desirable anti-inflammatory effect, helpful in controlling viral infection-related inflammation.

#### 2.2.3. Effect on Sub-Cellular Inflammatory Pathways (NF-kB, PI3K/Akt, and MAPK/JNK)

When a virus enters a host cell, the host cell recognizes its replication via pattern recognition receptors (PRRs) [109]. Virus RNA structure is involved in oligomerization of PRRs and activation of downstream transcription factors, in particular, interferon regulatory factors (IRF) and NF-κB. Activation of NF-κB and IRFs leads to engagement of cellular antiviral defense by the induction of type I and III interferons and chemokine secretion [110].

Chiou et al. investigated the effects of 8-prenylkaempferol (8-PK) in A549 cells infected with the influenza A (H1N1) virus. Results showed that interfering with the PI3K-Akt pathway is the main mechanism of 8-PK lead to protective effects against the influenza A virus. 8-PK decreased NF-κB and IRF-3 nuclear translocation through attenuation of Akt phosphorylation and PI3K activity. Finally, reduced production of regulated activation, normal T cell expressed and secreted (RANTES) through H1N1-infected A549 cells [111]. Zhu et al. represented that influenza A (H3N2) virus-induced autophagy in the A549 and Ana-1 infected cells via suppressing the mTOR signaling pathway. Baicalin could increase mTOR phosphorylation and rescued H3N2 virus effects in a dose-dependent manner [112]. In another study, baicalin was found to exert anti-influenza virus (H1N1-pdm09) activity by downregulation of the PI3k/Akt pathway caused through modulating viral protein NS1 expression [55]. Besides, biochanin A, an O-methylated isoflavone, indicated protective effects on H5N1 influenza A virus-infected cells via decreasing AKT, ERK1/2, JNK, and p38 phosphorylation. It could also modulate cellular signaling pathways, decrease IL-6, IL-8, CXCL10 (IP-10), TNF-α, and improved IκB levels [53].

CST represented inhibitory effects on the in vitro model of influenza A virus infection through inhibition of the NF-κB/p65 signal pathway, resulting in the downregulation of pro-inflammatory cytokines. CST also decreased phospho-p38 MAPK and phospho-JNK levels [59]. In another study by Ding et al., administration of hesperidin at the daily doses of 200 and 500 mg/kg for five days could inhibit pulmonary inflammation in influenza A virus (H1N1)-induced lung injury in rats. This effect was mediated via attenuating pro-inflammatory cytokine production, including IL-6 and TNF-α. Hesperidin also decreased IL-6 and TNF-α expression in H1N1 infected pulmonary microvascular endothelial cells through inhibition of MAPK signaling pathways [94]. Further, studies suggested ERK signaling pathway as a main modulator of the MAPK signaling pathway. Isorhamnetin (50 µM), a monomethoxyflavone, decreased ERK phosphorylation in MDCK cells after influenza A (H1N1) virus infection [113]. Jeong and colleagues investigated the cytotoxic effects of oroxylin A and tectorigenin in the CHME5 cells and primary human macrophages infected with HIV-1-D3. These flavonoids exert their effects via reducing the phosphorylation of PI3K, Akt, m-TOR, PDK1, GSK-3β, and Bad in the lipopolysaccharide/cycloheximide treated cells santin suppressed influenza A virus replication in the MDCK and THP-1 infected cells [114]. At the concentration of 60 µM, santin attenuated phosphorylation of p38 MAPK, ERK, JNK/SAPK, and NF-κB [61].

## 3. Discussion

**Figure 1:**
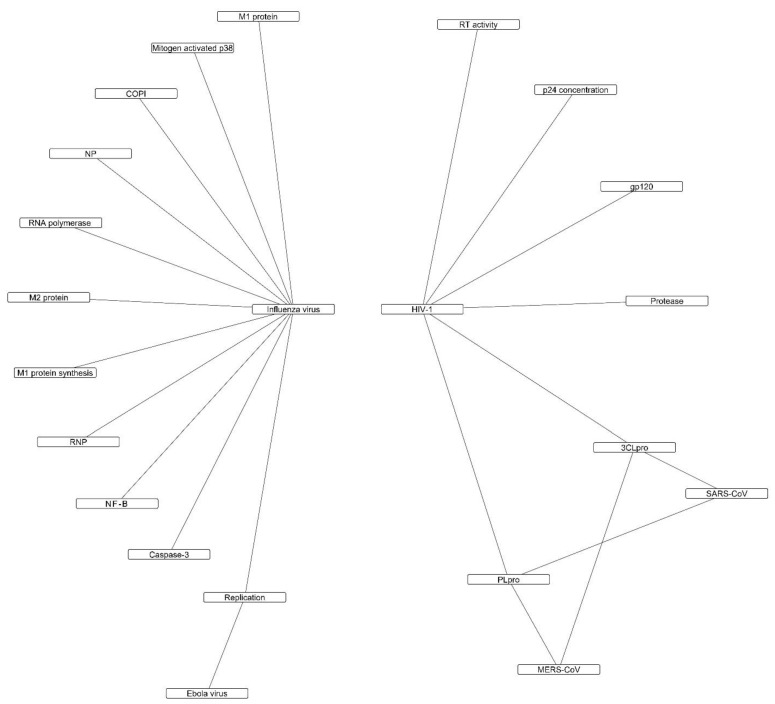
Direct antiviral mechanisms of flavonoids against viral infections with similar pathogenesis to SARS-CoV-2.

**Figure 2:**
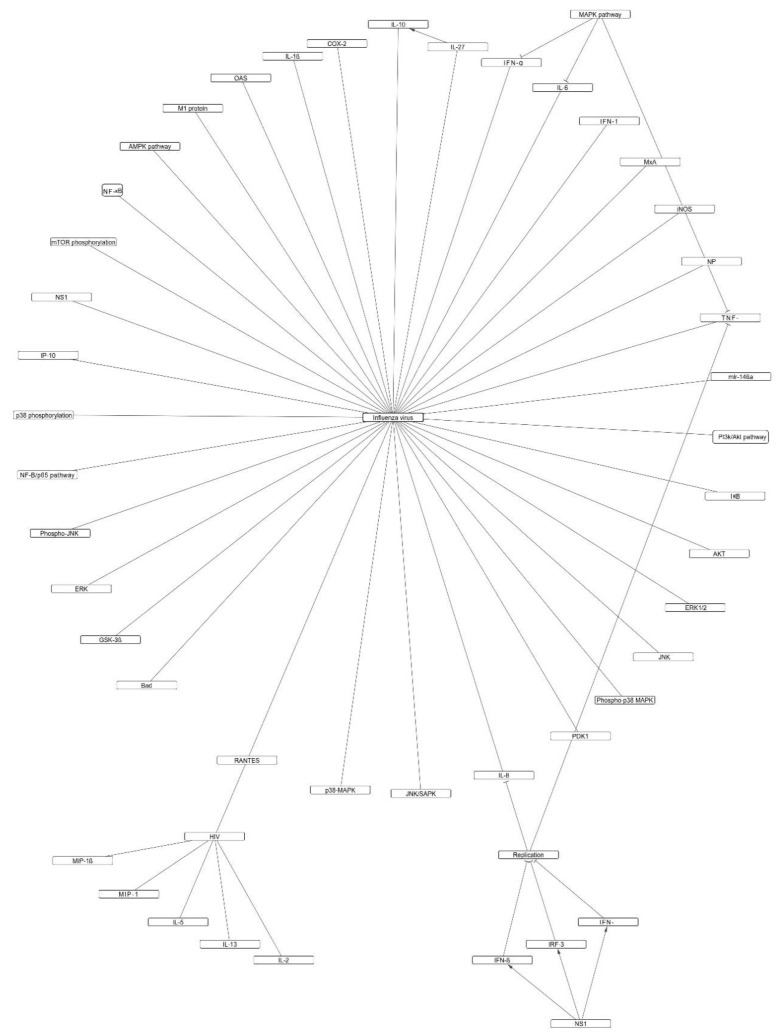
Indirect antiviral mechanisms of flavonoids against viral infections with similar pathogenesis to SARS-CoV-2.

Flavonoids as a class of safe and abundant phytoconstituents have attracted a lot of attention regarding their beneficial effects in COVID-19, and several attempts have been made to assess the structure-activity relationship of these compounds against SARS-CoV-2 proteins [115,116]. This paper reviewed the potential antiviral mechanisms of flavonoids based on the in vitro and in vivo studies on different viruses that follow the same pathogenic mechanisms as SARS-CoV-2, including HIV, influenza virus, ebola virus, SARS, and MERS. Available data on all virus and host targets were included in this study. Figure 1 and Figure 2 provide an overview of the direct and indirect mechanisms of flavonoids.

Amongst direct antiviral mechanisms, inhibition of viral proteases are the most frequently reported property of flavonoids. Due to the high similarity of SARS-CoV-2 proteases to those of SARS, flavonoids with inhibitory effects on these enzymes, such as isoliquiritigenin, kaempferol, and its derivatives, quercetin and its derivatives, theaflavins, flavonoids derived from *Angelica keiskei* (Miq.) Koidz. and *Broussonetia papyrifera* (L.) L’Hér. ex Vent. can be considered as candidates for future antiviral assessments against SARS-CoV-2 (Table 1). On the other hand, modulation of inflammatory host responses to the viral infections by the flavonoids seems to be the most important mechanism by which the complications of viral infection are managed. Baicalin and baicalein, biochanin A, cirsimaritin, gallocatechin-7-gallate, and hesperidin are flavonoids with modulating effects on both TNF-α and ILs and thus, can regulate severe conditions due to malfunction of host immune system such as cytokine storm.

According to the current literature, theaflavins, quercetin, luteolin, myricetin, kaempferol, catechins, hesperidin, and baicalin were the most promising flavonoids against the aforementioned viruses. Regarding the herbal sources of flavonoids, the most studied plants were *Camellia sinensis* (L.) Kuntze (tea) and *Scutellaria baicalensis* Georgi (skullcap). Green tea is a rich source of catechins, whereas black tea mostly contains theaflavins. Flavonoids from both types of tea have shown direct antiviral properties. Since tea is a popular drink in the human diet, it can be suggested as a safe dietary intervention for COVID-19 patients with mild to moderate symptoms. Due to its acceptable safety profile, tea can also be introduced as a suitable candidate for investigation in future clinical trials. Skullcap is a medicinal plant mostly used in Chinese medicine and is the natural source of baicalin, baicalein, oroxylin A, and wogonin. These flavonoids have demonstrated significant effects on the immune response of infected cells and animals via modulation of IFNs, endogenous antioxidant defense mechanisms, and inflammatory responses, as well as direct antiviral properties.

Some of the flavonoids reviewed in this study, such as cirsimaritin were shown to have antiviral activity higher than standard chemically synthesized drugs like ribavirin [59]. It should be mentioned that the results of in vitro antiviral studies do not necessarily guarantee the same potency and efficacy in clinical settings; though, they can be considered as a screening method to select the most effective compounds amongst numerous candidates for further in vivo and mechanistic evaluations. As previously mentioned, oseltamivir which is an anti-influenza agent has been designed and synthesized using shikimic acid, a plant-derived compound; thus, the introduced flavonoids in this review can be used as molecular backbones for the design and development of novel semisynthetic medicines with better bioavailability and clinical efficacy.

Despite hundreds of flavonoids evaluated against SARS-CoV-2 through virtual screenings, the experimental evidence on the in vitro or in vivo antiviral effect of these compounds against this exact type of virus is limited. Amongst the included flavonoids in our review, only four compounds, including baicalin, baicalein, quercetin, and isorhamnetin, were experimentally assessed in SARS-CoV-2-infected cells or animals.

Previous in silico studies and molecular analysis of different CoVs showed the potential antiviral effects of phytochemicals at different stages of viral biogenesis, including binding to ACE2, surface gangliosides, RdRp, viral spike protein, and viral protease in host cells and paved the way for more clinical and experimental studies [9,117,118,119,120,121,122,123]. Nevertheless, it should be considered that an acceptable antiviral activity in virtual screenings does not necessarily guarantee in vivo antiviral activity, and that is why an overview of flavonoids with antiviral properties in experimental studies is a further step toward the selection of natural antiviral agents. On the other hand, several of the mechanisms suggested for antiviral flavonoids in virtual screenings are not yet experimentally evaluated. In vitro and in vivo evidence discussed in this review, together with the results of virtual screenings, provides a better overview of the proper compounds for further investigations.

Additionally, there are some recently-published review articles that have focused on the effect of flavonoids on one specific target (e.g., ACE-2) or clinical manifestation (cytokine storms or lung injury) of SARS-CoV-2 infection [124,125,126,127]. Such points of view can put a focus on the development of natural medicines against one specific viral target; however, we preferred a more general approach in our study. We considered no limitation for antiviral/symptoms relieving mechanisms of flavonoids, and all experimental evidence of flavonoids on the above-mentioned viruses were included.

In conclusion, flavonoids can be considered as promising plant-derived compounds to manage SARS-CoV-2 infection via direct antiviral properties or management of host immune response to viral infection. Future experimental mechanistic and clinical studies are needed to further clarify the role of these compounds in primary and secondary prevention of SARS-CoV-2 infection.

## 4. Materials and Methods

Electronic databases, including PubMed, Scopus, and Web of Science, were searched from inception until April 2021 with the following formula: (COVID-19 OR SARS OR MERS OR corona OR HIV OR ebola OR influenza (title/abstract)) AND (plant OR extract OR herb OR phytochemical OR flavonoid (all fields)). As a supplementary search, the names of popular flavonoids including catechin, quercetin, rutin, hesperidin, hesperetin, naringenin, naringin, baicalin, bailaein, and epigallocatechin gallate (EGCG) were also individually searched in order to collect all related papers. After excluding duplicates, primary retrieved results were screened by two independent investigators based on the title and abstract. Selected papers were then checked based on their full text. Inclusion criteria were any in vitro or in vivo study in which the antiviral effect and mechanism of a flavonoid were evaluated. Studies on phytochemicals other than flavonoids, antiviral assessments of flavonoids without clarifying the mechanisms, and studies with non-English full-texts were excluded from our review. In silico studies were excluded unless coupled with an in vitro/in vivo experiment. We also did not discuss antiviral mechanisms such as inhibition of hemagglutinin and neuraminidase of influenza virus since these proteins are not mutual with SARS-CoV-2 and cannot be extrapolated to this virus. Those studies included in the final article are summarized in Table 1.

**Table 1:** Flavonoids with antiviral properties against SARS-CoV-2 and viral infections with similar pathogenesis.

Phytochemical Name	Plant Source	Model	Dose/Concentration	Mechanisms/Outcomes	Reference
(−)-epigallocatechin 3-*O*-gallate	*Limonium morisianum* Arrigoni	Anti-HIV-1 RT and IN	-	↓ HIV-1 RT-associated RNase	[128]
				H activity: IC50 = 0.21 μM	
				↓ IN catalytic function and IN-LEDGF-dependent activity: IC50 = 1.92 μM	
2′,4′-dihydroxy-6′-methoxy-3′,5′-dimethylchalcone	*Cleistocalyx operculatus* (Roxb.) Merr. and L.M.Perry	HEK293 cells infected with the plasmid H1N1 or oseltamivir-resistant novel H1N1 (H274Y)	20–40 μM	↑ cell viability,	[67]
		MDCK cells infected with influenza H1N1 A/PR/8/34 and H9N2 A/Chicken/Korea/O1310/2001		↓ NA activity (IC50 = 5.07 to 8.84 μM), viral replication, and CPE	
3-Deoxysappanchalcone	*Caesalpinia sappan* L.	MDCK, A549, and THP-1 cells infected with influenza virus A/PR/8/34 (H1N1)	30 µM	↓ viral genomic replication, DNA fragmentation, CCL5, CXCL10, IL-6, IL-1β, caspase 3/7, 8, and 9 activity and HA copy number (IC_50_ = 3.9 µM)	[68]
3-deoxysappanchalcone, Sappanchalcone and rhamnetin	*Caesalpinia sappan* L.	MDCK cells infected with influenza A/Guangdong/243/72 (H3N2), A/PR/8/34 (H1N1) and B/Jiangsu/10/2003	-	↓ NA activity: IC_50_ = 13.9–24.1 μg/mL ↓ CPE: IC_50_ = 1.06–15.4 μg/mL CC_50_ = 12.83–115.47 μg/mL SI = 6.23–16.27	[129]
5,7,4′-Trihydroxy-8-methoxyflavone	-	MDCK cells infected with influenza A/PR/8/34 (H1N1), A/Guizhou/54/89 (H3N2), and B/Ibaraki/2/85	-	↓ Sialidase activity:	[130]
				IC50 = 6.58–9.78 µg/mL	
6-hydroxyluteolin 7-*O*-β-d-glucoside	*Salvia plebeia* R.Br.	MDCK cells infected with influenza A/PR/8/34 (H1N1)	20, 50 μM	↓ NA activity and CPE, ↑ cell viability	[131]
8-Prenylkaempferol	*Sophora flavescens* Aiton	A549 cells infected with influenza A/PR/8/34 (H1N1) virus	1–30 μM	↓ RANTES production, NF-κB, IRF-3, PI3K activity, Akt phosphorylation, and IκB degradation	[111]
Agathisflavone	*Anacardium occidentale* L.	Mice infected with wild-type and oseltamivir-resistant influenza virus		IC50 = 20 to 2.0 µM, EC50 = 1.3 µM	[132]
				↓ NA activity and virus replication	
Apigenin 7-*O*-β-d-(4′-caffeoyl)glucuronide	*Chrysanthemum morifolium* Ramat.	MT-4 cells infected with HIV-I_IIIB_	-	↓ HIV-1 integrase activity:	[133]
				IC50 = 7.2 µg/mL	
				CPE:	
				EC50 = 41.86µg/mL,	
				SI ≥ 3.58	
Baicalein	-	A549 cells infected with influenza H5N1 virus strains (A/Thailand/1(Kan-1)/04 and A/Vietnam/1203/04)	40–100 µM	↓ viral nucleoprotein: IC50 = 18.79 µM	[53]
				CC50 = 109.41 µM	
				SI = 5.82	
		Primary human monocyte-derived macrophages (MDM) infected with influenza A/Thailand/1(Kan-1)/04		↓ virus titer, caspase-3 activation, NA activity,	
				↓ IL-6 and IL-8	
				↓ viral replication, IL6, CXCL10, and TNF-α	
Baicalin	-	Human lung epithelial A549 cells infected with influenza A/Jingfang/01/1986	20 μg/mL	↓ viral NP, M1 protein levels, viral titer, miR-146a expression, virus replication and viral copy number (EC50 = 17.04, 19.31 μg/mL), ↑ TRAF6 level, IFN-α, and IFN-β	[54]
		(H1N1) and A/Lufang/09/1993 (H3N2)		↓miR-146a expression and virus copy number	
		Balb/C mice inoculated intranasally with the influenza A H1N1 virus		↑ survival rate, IFN-α and IFN-β	
Baicalin	*Scutellaria baicalensis* Georgi	A549 and Ana-1 cells infected with influenza virus A3/Beijing/30/95 (H3N2)	12.5–50 μg/mL	↑ mTOR phosphorylation, ↓ autophagy, Atg5–Atg12 complex and LC3-II expression, EC_50_ = 15–15.6 µg/mL	[112]
Baicalin	-	A549 and MDCK cells infected with influenza virus A/H1N1/Eastern India/66/pdm09 (H1N1-pdm09)	0.5–320 µM	TD50 = 220 µM	[55]
				IC50 = 0.5 and 18 µM	
				↓ NP transcription, RIG-1, PKR, NS1 expression, viral replication, TNF-α, IL-8, p-85b–NS1 binding, p-Akt, M1 protein, ↑ IRF-3, IFN-γ, and IFN-β	
		BALB/c mice infected intranasally with H1N1-pdm09	(10–120 mg/kg/day) twice daily for 3 days	↓ viral titer:	
				MIC50 ≈ 80 mg/kg/day	
				↓ p-Akt and M1 protein expression	
Baicalin	*Scutellaria baicalensis* Georgi	MDCK and A549 cells infected with influenza A/FM1/1/47 (H1N1) and A/Beijing/32/92 (H3N2)	20–80 µg/mL (in MDCK cells)	↑ cell viability, ↓ virus replication, and CPE:	[71]
				EC50 = 40.3 and 104.9 µg/mL	
			5–40 µg/mL (in A549 cells)	SI: 2.1–8.6	
		ICR mice infected with influenza A/FM1/1/47 (H1N1) virus		↓ NA activity:	
			50–200 mg/kg/d for 5 days, i.v injection	IC50 = 52.3 and 85.8µg/mL	
				↓ death rate, weight loss, ↑ mean day to death, survival rates, and improved the lung parameters	
Baicalin	*Scutellaria baicalensis* Georgi	Hos/CD4/CCR5 or Hos/CD4/CXCR4 cells infected with recombinant vaccinia virus vTF7-3	0.04 to 400 µM	↓ X4 and R5 HIV-1 Env-mediated fusion, CAT activity, CD4/CXCR4, CD4/CCR5, and HIV-1 entry	[134]
Baicalein, Baicalin	*-*	Vero CCL-81 cell line infected with SARS-CoV-2	20 µM	↓ viral RdRp and viral replication	[49]
				CC50 = 86–100 µM	
				EC50 = 1–9 µM	
Baicalein	-	Vero E6 cells infected with SARS-CoV-2	0.1 μM	↓ body weight loss, the replication of the virus, relieved the lesions of lung tissue, inflammatory cell infiltration, IL-1β and TNF-α	[66]
		hACE2 transgenic mice infected with SARS-CoV-2	200 mg/kg	↑ respiratory function	
Biochanin A	-	A549 cells infected with influenza H5N1 virus strains (A/Thailand/1(Kan-1)/04 and A/Vietnam/1203/04)	40 µM	↓ viral nucleoprotein: IC50 = 8.92 µM	[53]
				CC50 = 49.91 µM	
				SI = 5.60	
		Primary human monocyte-derived macrophages (MDM) infected with influenza A/Thailand/1(Kan-1)/04		↓ virus titer, caspase-3 activation, NFκB p65 accumulation, IL-6, IL-8, CXCL10 production, phosphorylation of AKT and ERK 1/2 and ↑ IκB levels	
				↓ IL6, CXCL10, and TNF-α	
Catechin	-	A549 cells infected with influenza A H1N1	5–50 μM	↓ NA and HA activity, viral load, and virus-induced autophagy	[135]
Catechins	*Camellia sinensis* (L.) Kuntze	MDCK cell infected with influenza A/Chile/1/83 (H1N1), A/Sydney/5/97 (H3N2), and B/Yamagata/16/88	30–1200 µM	↓ plaque formation:	[136]
				EC50 = 22.2–318 µM	
				↓ NA activity, HA activity, and viral RNA synthesis	
Catechins (EGCG, ECG and C5G)	*Camellia sinensis* (L.) Kuntze	MDCK cells infected with influenza A/Victoria/503/2013,	50–100 µM	↓ NA activity	[137]
				IC50 = 100.3–173 µM	
		A/SouthAustralia/21/2013 and A/Perth/25/2013		CC50 = 274–551.3 µM	
				↓ plaque number	
				EC50 = 28.4–34.3 µM	
Catechins with a galloyl moiety	-	HIV-1 integrase assay kit	0.1–100 μM	↓ HIV-1 integrase activity IC_50_ = 0.56–3.02 μM	[138]
Cirsimaritin	-	MDCK and THP-1 cells infected with influenza A/Fort Monmouth/1/1947(H1N1), A/tianjinjinnan/15/2009(H1N1) and A/JiangXi/312/2006(H3N2)	2.5–20 μg/mL	↓ CPE:	[59]
				IC50 = 5.8, 6.3, 11.1 μg/mL, SI = 24.3, 26.4, 13.8, and TC50 = 153.3	
				↓ viral replication, M2 viral protein expression, intracellular	
				p65/NF-κB protein, p65/NF-κB phosphorylation, TNF-α, IL-1β,	
				IL-8, IL-10, COX-2 expression, phospho-p38 MAPK, and ↓ phospho-JNK	
C-methylated flavonoids	*Cleistocalyx operculatus* (Roxb.) Merr. and L.M.Perry	MDCK and 293T cells infected with influenza A/PR/8/34 (H1N1), A/Chicken/Korea/O1310/2001 (H9N2), A/Sw/Kor/CAH1/04 (H1N1, KCTC 11165BP), novel H1N1 (WT) and Oseltamivir-resistant novel H1N1 (H274Y)	-	↓ CPE:	[139]
				EC50 = 4.90–8.79 µM	
				SI = 10.15 to > 24.49	
				↓ NA activity:	
				IC50 = 2.55–93.77 µM	
EGCG	*Camellia sinensis* (L.) Kuntze (green tea)	MDCK cells infected with influenza A/Puerto Rico/8/34 (H1N1) (PR8), A/Hong Kong/8/68 (H3N2) (HK), A/Brisbane/59/2007 (H1N1) (BB), A/Taiwan/1/1986 (H1N1) (TW), A/Korea/01/2009 (H1N1) (KR) and B/Panama/45/1990 (PNM)	10–100 µM	↓ CPE:	[140]
				EC50 = 8.9–17.3 µM	
				↓ hemifusion events, viral membrane integrity, cell penetration capacity, NP protein, viral entry, and NA activity: IC50 = 133.2 to > 500 µM	
EGCG	-	PBMCs, CD4^+^ T cells and macrophages infected with several clinical isolates of HIV-1	6–100 μM	↓ HIV-1 p24 antigen: IC50 = 4.5–12 μM	[141]
				↓ HIV-1 infectivity,	
				HIV-1–glycoprotein 120 attachment to the CD4 molecule,	
				CC50 > 100 μM	
EGCG	-	CD4^+^ T cells	0.2–20 μM	↓ HIV-1 gp 120 binding to the CD4^+^ T cells	[142]
EGCG	*Camelia sinensis* (L.) Kuntze	Peripheral blood CD4^+^ T cells (by flow cytometry)	25–100 µM	↓ CD4 expression, anti-CD4 antibody binding to its antigen, gp120 binding to CD4, and HIV infection	[143]
EGCG	*Camelia sinensis* (L.) Kuntze	Peripheral blood lymphocytes infected with either LAI/IIIB or Bal HIV strains	1–50 µM	↓ virus replication, RT activity, and p24	[75]
EGCG	*Camelia sinensis* (L.) Kuntze	THP-1 and H9 cells infected with HIV-1	1–100 µM	↓ RT activity, protease activity, p24, viral entry, and viral production	[40]
Flavonoid aglycones (demethoxymatteucinol, matteucinol, matteucin, methoxymatteucin, and 3′-hydroxy-5′-methoxy-6,8-dimethylhuazhongilexone)	*Pentarhizidium orientale* (Hook.) Hayata	MDCK cells infected with influenza A/PR/8/34 (H1N1) or A/chicken/Korea/01210/2001 (H9N2)	-	↓ NA activities and CPE:	[144]
				IC50 = 23.1–31.3 μM, EC50 = 21.4–30.7 μM, CC50 = 77.6 μM (demethoxymatteucinol), CC50 => 100 μM (matteucin, methoxymatteucin, and 3′-hydroxy-5′-methoxy-6,8-dimethylhuazhongilexone)	
Flavonoid compounds	-	SARS-CoV proteases (recombinant 3CL^pro^) expressed in *Pichia pastoris* GS115	200 µM	↓ 3CLpro activity:	[145]
				IC50 = 47–381 µM	
Gallocatechin-7-gallate	*Pithecellobium clypearia* Benth	A549 and MDCK cells infected with influenza A/PR/8/34 (H1N1)	3–30 μM	↓ CPE:	[58]
				CC50 = above 100 μM, EC50 = 1.69 μM	
				↓ NP and M2 expression levels, HA mRNA expression, M2/M1 levels, and phosphorylation of SF2/ASF and SC35	
		ICR mice infected intranasally with influenza A H1N1 virus	30 mg/kg/d, i.v, for 5 days	↓ viral NP mRNA expression, TNF-α, IL-1β, IL-6, bodyweight loss, acute lung injury and lung virus titer, ↑ survival rate, T-lymphocyte stimulation index, B-lymphocyte stimulation index, and spleen and thymus indices	
Genistein	-	Heterologously expression of viral protein U of HIV in Xenopus oocyte	20 µM	↓ Ba2+-sensitive current and blocked Vpu ion channels	[146]
Genistein	*-*	Primary human macrophages infected with HIV-1_Ba-L_ Env expressed on 293 T cells	5–10 µg/mL	↓ R5 Env pseudotyped virus infection, HIV-1Ba-L Env expressing cells and macrophages cell-fusion, reporter gene expression, virus penetration, gp120-induced TNF-α secretion, virus replication	[76]
Ginkgetin	*Ginkgo biloba* L. and *Cephalotaxus harringtonia* K. Koch	MDCK cells infected with influenza A/PR/8/34 (H1N1), A/Guizhou/54/89 (H3N2), and B/Ibaraki/2/85	-	↓ sialidase activity:	[130]
				IC50 = 9.78 to > 100 µg/mL	
Herbacitrin	*Drosera peltata* Thunb.	HIV-1 infected MT-4 and MT-2 cell culture	21.5 µM	↓ HIV-1 replication, HIV-1 RT activity, IN activity, and p24 level	[77]
Hesperidin	-	R5-type HIV-1 in CD4^+^ NKT cells and human Vδ1^+^ cells in PBMCs	30–100 µg/mL	↑ IL-2, IL-5, IL-13, MIP-1α, MIP-1β, RANTES, CFSE, and CD25 expression, and ↓ viral replication	[104]
Hesperidin	-	Influenza A virus (H1N1) induced lung injury in male Sprague-Dawley rats, by the intrathecal route	200 and 500 mg/kg/d, i.p., for 5 days	↑ pulmonary function,	[94]
				↓ Local numbers of immune cells, TNF-α, IL-6, and IFN-α	
		H1N1 infected pulmonary microvascular endothelial cells	1 mg/mL	↓ TNF-α, IL-6, IFN-α, phosphorylated p38 and JNK	
Hexamethoxyflavone (5-Hydroxy-3,6,7,8,3′,4′-hexamethoxyflavone)	*Marcetia taxifolia* (A. St.-Hil.) DC.	MT4 cells infected with HIV-1 (HTLV-IIIB/H9)	45 µM	↓ HIV-1 RT activity: IC50 = 4.1 µM,	[147]
				EC50 = 0.04 μM, CC50 > 50 μM	
Hispidulin	*Salvia plebeia* R. Br.	MDCK cells infected with influenza strain H1N1 A/PR/8/34 virus	20–50 μM	↓ CPE and NA activity:	[148]
				IC50 = 19.83 μM, EC50 = 22.62 μM, SI > 8.90	
				↑ cell survival rate recovered the chromosome condensation	
Homoplantaginin	*Salvia plebeia* R.Br.	MDCK cells infected with influenza A/PR/8/34 (H1N1)	20, 50 μM	↓ NA activity and CPE, ↑ cell viability	[131]
IND02	*Cinnamomum zeylanicum* Blume	MAGI cells and PBMCs infected with HIV-1 LAI and NL4-3	5–30 μM	↓ gp120 binding to HS (IC_50_ = 7 μM), gp120 binding to CD4 (IC_50_ = 20 μM), and envelope binding to CD4	[149]
IND02-trimer	*Cinnamomum zeylanicum* Blume	MAGI cells and PBMCs infected with HIV-1 LAI, NL4-3, Ba-L and clinical isolates (HIV-1 92UG029(A-X4), HIV-1 92HT599 (B-X4), HIV-1 96USHIPS4 (B-X4/R5) and HIV-1 98IN017 (C-X4))	2–20 μM	↓ gp120 binding to HS (IC50 = 7.5 μM), EC50 = 0.8–7 μM, CC50 = 96 and 23	[149]
		HIV-1-infected CD4+ and CD8+ T cells	0.46–46.3 μM	↓ up-modulation of Tim-3 and PD-1	
Isoliquiritigenin	-	SARS-CoV proteases (3CL^pro^ and PL^pro^) expressed in *E. coli* BL21	-	↓ PLpro activity: IC50 = 24.6 µM, Deubiquitination activity = 17.2, DeISGylation activity = 12.6,	[37]
				↓ 3CLpro activity:	
		MERS-CoV proteases (3CL^pro^ and PL^pro^) expressed in *E. coli* BL21		IC50 = 61.9 µM	
				↓ PLpro activity: IC50 = 82.2 µM,	
				↓ 3CLpro activity: IC50 = 33.9 µM	
Isoquercetin	-	MDCK or Vero cells infected with influenza A viruses from pigs (A/swine/OH/511445/2007 [H1N1], Oh7) and human (A/PR/8/34 [H1N1], PR8), and human influenza B virus (B/Lee/40)	1–5 µM	↓ viral replication and CPE:	[79]
				ED50 = 1.2 µM	
			2–10 mg/kg/day, i.p.	TD50 = 45 µM	
		BALB/c mice infected with influenza A/PR/8/34, H1N1virus		SI = 38	
				↓ IFN-γ, iNOS, RANTES, virus titers, viral bronchitis, and bronchiolitis	
Isorhamnetin	-	MDCK cells infected with influenza virus A/PR/08/34 (H1N1)Embryonated chicken eggs infected with influenza virus A/PR/08/34 (H1N1)	50 µM	↑ cell viability	[113]
				EC50 = 23 µM	
				CC50 > 280 µM	
				SI > 12	
		C57BL/6 mice infected with influenza A/PR/8/34 (H1N1)	1 mg/kg/day for 5 days (intranasal route)	↓ autophagy, ROS generation, ERK phosphorylation, cytoplasmic lysosome acidification, NA and HA expression, and NA activity	
				↓ virus titer, adsorption onto RBCs and RBCs hemolysis	
				↓ lung virus titer and body weight loss,	
				↑ survival rate	
Quercetin	*Elaeagnus rhamnoides* (L.) A.Nelson (synonym: *Hippophae rhamnoides* L.)	ACE2h cells infected with SARS-CoV-2	50 µM	↓ Viral entry	[65]
Isorhamnetin				↓ Viral Binding affinity to ACE2	
Kaempferol	-	SARS-CoV proteases (3CL^pro^ and PL^pro^) expressed in *E. coli* BL21	-	↓ PLpro activity: IC50 = 16.3 µM, Deubiquitination activity = 61.7, DeISGylation activity = 71.7,	[37]
				↓ 3CLpro activity:	
		MERS-CoV proteases (3CL^pro^ and PL^pro^) expressed in *E. coli* BL21		IC50 = 116.3 µM	
				↓ PLpro activity: IC50 = 206.6 µM,	
				↓ 3CLpro activity: IC50 = 35.3 µM	
Kaempferol derivatives	-	Heterologously expression of 3a protein of SARS-CoV in Xenopus oocyte	10–20 µM	↓ Ba^2+^-sensitive current and 3a-mediated current, blocked 3a-protein channel IC_50_ = 2.3 µM (for juglanin)	[36]
Linarin	-	R5-type HIV-1 in CD4^+^ NKT cells and human Vδ1^+^ cells in PBMCs	10–100 µg/mL	↑ IL-2, IL-5, IL-13, MIP-1α, MIP-1β, RANTES, CFSE, and CD25 expression, ↓ viral replication	[104]
Luteolin	-	MDCK, Calu-3, and Vero cells infected with influenza A/Jiangxi/312/2006 (H3N2) and A/Fort Monmouth/1/1947 (H1N1)	3.75–240 μM	↓ CPE:	[60]
				IC50 = 6.89, 7.15 μM, CC50 = 148–240 μM	
				↓ M2 viral protein expression, virus absorption, and internalization	
Luteolin	*Salvia plebeia* R. Br.	MDCK cells infected with influenza strain H1N1 A/PR/8/34 virus	50 μM	↓ NA activity: IC_50_ = 17.96 μM, EC_50_ = cytotoxic	[148]
Luteolin	-	PBMCs, TZM-bl reporter, and Jurkat cellsinfected with wild–type HIV (NLENY1) or VSV-HIV-1	5–10 µM	↓ clade-B- and -C -Tat-driven LTR transactivation, reactivation of latent HIV-1 infection, HIV-1 gene expression, LTR activity, PBMC cell aggregation/syncytia, viral entry	[150]
Luteolin and luteolin 7-methyl ether	*Coleus parvifolius* Benth.	MT-4 cells infected with HIV-1 (HTLV III_B_)	-	↓ HIV-1 integrase activity:	[151]
				IC50 = 11–70 µM	
				↓ viral replication	
Myricetin	-	TZM-bl cell infected with HIV-1 BaL (R5 tropic), H9 and PBMC cells infected with HIV-1 MN (X4 tropic), and the dual tropic (X4R5) HIV-1 89.6,	0.01–100 μM	↓ p24 antigen:	[152]
				IC50 = 1.76–22.91 μM, CC50 = 804.94–1214.72 μM,	
		Anti-HIV-1 RT		↓ HIV-1 RT: IC50 = 203.65 μM	
Myricetin	-	TZM-bl cell infected with HIV-1 BaL (R5 tropic), H9 and PBMC cells infected with HIV-1 MN (X4 tropic), and the dual tropic (X4R5) HIV-1 89.6,	0.01–100 µM	↑ cell viability,	[152]
				↓ p24 antigen:	
				IC50 = 1.76–22.91 μM, CC50 = 804.94–1214.72 μM,	
				↓ HIV-1 RT:	
		Anti-HIV-1 RT		IC50 = 203.65 μM	
Myricetin-3-*O*-(6″-*O*-galloyl)-β-D-galactopyranoside	*Limonium morisianum* Arrigoni	Anti-HIV-1 RT and IN	-	↓ HIV-1 RT-associated RNase	[128]
				H activity: IC50 = 10.9 μM	
				↓ IN catalytic function and IN-LEDGF-dependent activity: IC50 = 6.47 μM	
Myricetin derivatives	*Marcetia taxifolia* (A. St.-Hil.) DC.	MT4 cells infected with HIV-1 (HTLV-IIIB/H9)	-	↓ HIV-1 RT activity: IC_50_ = 7.6–13.8 µM, EC_50_ = 45–230 μM, SI > 1.3–7	[153]
Myricetin-3′,5′-dimethylether 3-*O*-*β*-D-galactopyranoside	*Cleistocalyx operculatus* (Roxb.) Merr. and L.M.Perry	HEK293 cells infected with the plasmid H1N1 or oseltamivir-resistant novel H1N1 (H274Y)	40 μM	↑ cell viability,	[67]
		MDCK cells infected with influenza H1N1 A/PR/8/34 and H9N2 A/Chicken/Korea/O1310/2001		↓ NA activity (IC50 = 6.50 to 9.34 μM), viral replication and CPE	
Naringenin	*-*	Vero E6 cells infected with HCoVOC43, HCoV229E, and SARS-CoV-2	62.5, 250 μM	↓ TPC2, CPE activity	[74]
Nepetin	*Salvia plebeia* R. Br.	MDCK cells infected with influenza strain H1N1 A/PR/8/34 virus	20–50 μM	↓ CPE and NA activity:	[148]
				IC50 = 11.18 μM, EC50 = 17.45 μM, SI = ~11.47,	
				↑ cell survival rate	
Nepitrin	*Salvia plebeia* R.Br.	MDCK cells infected with influenza A/PR/8/34 (H1N1)	20, 50 μM	↓ NA activity and CPE, ↑ cell viability	[131]
Oroxylin A	-	MDCK and A549 cells infected with influenza A/FM/1/47 (H1N1), A/Beijing/32/92 (H3N2), and oseltamivir-resistant	40–50 μM	↓ CPE:	[52]
				IC50 = 270.9, 245.0, 241.4 μM, EC50 = 44.6, 36.1, 109.4 μM	
		A/FM/1/47-H275Y (H1N1-H275Y) viruses H1N1-H275Y and A/Anhui/1/2013-R294 K (H7N9-R294 K)	100 μM	↓ viral mRNA and M1 protein expression	
				↓ NA activity, IC50 = 241.4 and 203.6 μM	
		ICR mice infected intranasally with the A/FM/1/47 (H1N1)	100 mg/kg/d, p.o.	↑ IFN-β, IFN-γ and survival rate ↓ body weight loss, lung injury, lung indexes and lung scores	
Oroxylin A	*Scutellaria baicalensis* Georgi	CHME5 cells and primary human macrophages infected with HIV-1-D3	5–20 µM	↓ phosphorylation of PI3K, PDK1, Akt, activation of GSK3β, m-TOR, and Bad	[114]
Pentamethoxyflavone(5,3′-dihydroxy-3,6,7,8,4′-pentamethoxyflavone)	*Marcetia taxifolia* (A. St.-Hil.) DC.	MT4 cells infected with HIV-1 (HTLV-IIIB/H9)	45 µM	↓ HIV-1 RT activity: IC_50_ = 0.4 µM, EC_50_ = 0.05 μM, CC_50_ > 50 μM	[154]
Pongamones A–E	*Pongamia pinnata* (L.) Pierre	In vitro inhibitory activity against HIV-1 RT	-	↓ RT activity	[155]
				IC50 > 10 µg/mL	
Prenylisoflavonoids	*Erythrina senegalensis* DC.	In vitro inhibitory activity against recombinant HIV-1 protease	-	↓ HIV-1 protease activity	[44]
				IC50 = 0.5–30.1 µM	
Purified chalcones	*Angelica keiskei* (Miq.) Koidz.	SARS-CoV proteases (3CL^pro^ and PL^pro^) expressed in *E. coli* BL21	-	↓ 3CLpro activity:	[156]
				Cell-free cleavage: IC50 = 11.4–129.8 µM,	
				Cell-based cleavage: IC50 = 5.8–50.8 µM, SI = 0.4–9.2	
				↓ PLpro activity: IC50 = 1.2–46.4 µM, Deubiquitination activity = 2.6–44.1, DeISGylation activity = 1.1–11.3	
Purified flavanone glucosides	*Thevetia peruviana* SCHUM.	HIV-1 IN protein expressed in *E. coli*,		↓ HIV-1 RDDP activity: IC50 = 20–43 µM	[157]
		RDDP and DDDP inhibitory activity assay		↓ HIV-1 DDDP activity: IC50 = 42 and 69 µM	
				↓ HIV-1 IN activity: IC50 = 5–45 µM	
Purified flavones	*Kaempferia parviflora* Wall. ex Baker	In vitro inhibitory activity against HIV-1 protease	-	↓ HIV-1 protease	[158]
				IC50 = 19.04–160.07 µM	
Purified flavonoids	*Pithecellobium clypearia* Benth	A549 cells infected with influenza A/PR/8/34 (H1N1), A/Sydney/5/97 (H3N2) and B/Jiangsu/10/2003	3–30 μg/mL	↓ NA activity:	[105]
				IC50 = 29.77–39.15 µg/mL	
				↓ IL-6 and MCP-1	
Purified flavonoids	*-*	NAs from influenza A/PR/8/34 (H1N1), A/Jinan/15/90 (H3N2), andB/Jiangshu/10/2003	-	↓ NA activity:	[159]
				IC50 = 22–87.6 μM	
				↓ CPE:	
		MDCK cells infected with influenza A/Jinan/15/90 (H3N2)		IC50 = 4.74–24.70 µM	
				SI = 1.82–9.64	
Purified flavonoids	*Elsholtzia rugulosa* Hemsl.	NAs from influenza viruses A/PR/8/34(H1N1), A/Jinan/15/90(H3N2) and B/Jiangsu/10/2003	-	↓ NA activity:	[160]
				IC50 = 7.81–28.49 μM	
				↓ viral replication and CPE:	
		MDCK cells infected with influenza A/Jinan/15/90 (H3N2)		IC50 = 1.43 to > 500 µM,	
				SI = 1.73–7.48	
Purified flavonoids (Ochnaflavone 7″-O-methyl ether and 2″,3″dihydroochnaflavone 7″ methyl ether)	*Ochna integerrima* (Lour.) Merr.	1A2 cell line infected with ^∆Tat/rev^MC99 virus	200 µg/mL	↓ RT activity:	[161]
				IC50 = 2.0 and 2.4 µg/mL	
				↓ HIV-1 activities:	
				EC50 = 2 and 0.9 µg/mL	
				IC50 = 6.3 and 2.9 µg/mL	
				SI = 3.1 and 3.2	
Purified flavonol glycosides	*Zanthoxylum piperitum* (L.)	MDCK cells infected with influenza A/NWS/33 (H1N1)	7.8–1000 μg/mL	↓ NA activity:	[162]
				IC50 = 211–434 μg/mL	
				↓ PFU	
Purified flavonols	*Rhodiola rosea* L.	MDCK cells infected with influenza A/PR/8/34 (H1N1) and A/Chicken/Korea/MS96/96 (H9N2)	-	↓ CPE:	[163]
				EC50 = 6.25–145.4 µM	
				SI = 1.6 to > 48	
		Recombinant influenza A virus H1N1(rvH1N1)		↓ NA activity:	
				IC50 = 2.2–56.9 μg/mL	
Purified flavonoids	*Broussonetia papyrifera* (L.) L’Hér. ex Vent.	SARS-CoV proteases (3CL^pro^ and PL^pro^) expressed in *E. coli* BL21	-	↓ PLpro activity: IC50 = 3.7–66.2 µM, Deubiquitination activity = 7.6–74.8, DeISGylation activity = 8.5–70.8,	[37]
				↓ 3CLpro activity:	
				IC50 = 30.2–233.3 µM	
		MERS-CoV proteases (3CL^pro^ and PL^pro^) expressed in *E. coli* BL21		↓ PLpro activity: IC50 = 39.5–171.6 µM,	
				↓ 3CLpro activity: IC50 = 27.9–193.7 µM	
Quercetin	-	SARS-CoV proteases (3CLpro and PLpro) expressed in *E. coli* BL21	-	↓ PLpro activity: IC50 = 8.6 µM, Deubiquitination activity = 20.7, DeISGylation activity = 34.4,	[37]
				↓ 3CLpro activity:	
		MERS-CoV proteases (3CLpro and PLpro) expressed in *E. coli* BL21		IC50 = 52.7 µM	
				↓ 3CLpro activity: IC50 = 34.8 µM	
Kaempferol		Vero E6 cells infected with SARS-CoV	125, 62.5, and 31.25 μM	↓ Virus-induced cell death, 3CLprotease	[35]
Quercetin	-	MDCK and A549 cells infected with influenza A/Puerto Rico/8/34 (H1N1), A/FM-1/47/1 (H1N1) and A/Aichi/2/68 (H3N2)	12.5–100 µg/mL (50 µg/mL)	↓ CPE:	[62]
				IC50 = 2.738–7.756 µg/mL, IC90 = 8.24–24.58 µg/mL,	
				↓ HA mRNA transcription, viral NP protein synthesis, viral HA expression and virus infection rate, target the membrane fusion process during virus entry	
Quercetin	-	Inhibitory activity against recombinant HIV-1 protease	-	↓ HIV-1 protease activity:	[45]
				IC50 = 58.8 µM	
Quercetin 3-O-(6″-feruloyl)-β-D-galactopyranoside	*Polygonum viscosum* Buch.-Ham. ex D. Don	In vitro anti-HIV-1 activity	-	↓ RT activity	[164]
				IC50 = 25.61 µg/mL	
Quercetin 3-rhamnoside	*Houttuynia cordata* Thunb.	MDCK cells infected with influenza A/WS/33	10–100 µg/mL	↓ CPE, viral mRNA synthesis, virus replication and virus infection	[80]
Quercetin 3-β-O-D-glucoside	-	Vero E6 epithelial	10 μM	↓ virus replication (EC_50_ = 5.3 µM, EC_90_ = 9.3 µM), viral titers and entry of Ebola viruses	[81]
		cells infected with EBOV-Kikwit-GFP, EBOV-Makona			
		and SUDV or VSV-EBOV, and VSV-SUDV	50 mg/kg every other day, i.p.	↓ virus replication and body weight loss,	
		BALB/c or C57BL/6 mice infected with mouse-adapted Ebola virus		↑ survival rate	
Quercetin-3-O-α-L-rhamnopyranoside	*Rapanea melanophloeos* (L.) Mez	MDCK cells infected with A/Puerto Rico/8/1934 (H1N1)	150 μg/mL	↓ CPE:	[106]
				CC50 = 200 μg/mL, EC50 = 25 μg/mL, EC90 = 100 μg/mL	
				↓ NP and M2 genes copy numbers, viral titer, HA titer and TNF-α,	
				↑ IL-27 protein level and cell viability	
Quercetin-7-O-glucoside	*Dianthus superbus var. longicalycinus* (Maxim.) F.N.Williams	MDCK cells infected with influenza A/Vic/3/75 (H3N2), A/PR/8/34 (H1N1), B/Maryland/1/59 and B/Lee/40 viruses	10 μg/mL	↓ CPE:	[50]
				IC50 = 3.10 μg/mL to 8.19 μg/mL, CC50 > 100 μg/mL, SI = 12.21 to 32.25,	
				↓ ROS, autophagy, viral RNA synthesis and viral RNA polymerase	
Quercetin-β-galactoside	-	SARS-CoV proteases (3CL^pro^ and PL^pro^) expressed in *E. coli* BL21	-	↓ PLpro activity: IC50 = 51.9 µM, Deubiquitination activity = 136.9, DeISGylation activity = 67.7,	[37]
				↓ 3CLpro activity:	
				IC50 = 128.8 µM	
		MERS-CoV proteases (3CL^pro^ and PL^pro^) expressed in *E. coli* BL21		↓ PLpro activity: IC50 = 129.4 µM,	
				↓ 3CLpro activity: IC50 = 68.0 µM	
Santin	*Artemisia rupestris* L.	MDCK and THP-1 cells infected with influenza strain A/Fort Monmouth/1/1947 (H1N1) and A/Wuhan/359/1995	60 µM	↓ CPE:	[61]
				IC50 = 27.68, 37.20 μM, SI = 14.45, 10.75, TC50 > 400 μM	
				↓ M2 viral protein expression, phosphorylation of p38 MAPK,	
		(H3N2)		JNK/SAPK, ERK, NF-κB, TNF-α, IL-1β, IL-6, IL-8, and IL-10 production	
Scutellarin	*Erigeron breviscapus* (Vant.) Hand.-Mazz	C8166 cells infected with HIV-1_IIIB_ and HIV-1_IIIB_/H9,	54–541 µM	↓ HIV-1 replication:	[165]
		MT-2 cells infected with HIV-1_74V_,		EC50 = 15–253 µM	
		PBMC cells infected with HIV-1_KM018_		CC50 = 336 -> 1082 µM	
		Purified recombinant HIV-1 RT		↓ RT activity, HIV-1 particle attachment and fusion	
Tectorigenin	*Pueraria thunbergiana* (Siebold and Zucc.) Benth.	CHME5 cells and primary human macrophages infected with HIV-1-D3	5–20 µM	↓ phosphorylation of PI3K, PDK1, Akt, activation of GSK3β, m-TOR, and Bad	[114]
Theaflavins	*Camellia sinensis* (L.) Kuntze (black tea)	MDCK andA549 cells infected with influenza A/PR/8/34(A/H1N1), A/Sydney/5/97(A/H3N2) and B/Jiangsu/10/2003	0.1 to 30 µg/mL	↓ NA:	[108]
				IC50 = 10.67–49.6 µM	
				↓ CPE:	
				CC50 = 76.7–177.1 µM	
				↓ HA activity, IL-6 and vRNP nuclear localization,	
Theaflavins	-	SARS-CoV proteases (3CL^pro^) expressed in *E. coli*	-	↓ 3CLpro activity	[43]
				IC50 = 3–9.5 µM	
Tricin	-	MDCK cells infected with influenza A/Solomon islands/3/2006 (H1N1), A/Hiroshima/52/2005 (H3N2), A/California/07/2009 (H1N1pdm), A/Narita/1/2009 (H1N1pdm) and B/Malaysia/2506/2004	3.3–30 µM	↓ HA and matrix protein, mRNA expression, virus titer (EC50 = 3.4–10.2 µM)	[63]
		DBA/2 Cr mice infected intranasally with influenza A/PR/8/34 virus	20–100 µg/kg, p.o.	↓ Body weight loss,	
				↑ Survival rate	
Wogonin	*Scutellaria baicalensis* Georgi	MDCK and A549 cells infected with human influenza virus A/Puerto-Rico/8/34 (H1N1) PR8, seasonal H1N1, H3N2 and B (yamagata lineage)	10 μg/mL	↓ NA and NS1 levels, viral replication, Akt phosphorylation, ↑ IFN-β, IFN-λ1, MxA, OAS, AMPK phosphorylation, phospho-IRF-3 expression, cleaved PARP, and caspase-3 expression and apoptosis	[95]
				↓ Plaque formation: IC50 = 10 μg/mL	

**Abbreviations:** HA: hemagglutinin; NA: neuraminidase; MDCK: Madin-Darby canine kidney; HIV: human immunodeficiency virus; CD: cluster of differentiation; NKT: natural killer T cells; CPE: cytopathic effect; IC50: inhibitory concentration 50%; CC50: cytotoxic concentration 50%; THP-1: Human acute monocytic leukemia; SI: selectivity index; TC50: 50% toxicity concentration; MAPK: Mitogen-activated protein Kinase; JNK: c-Jun N-terminal kinase; SAPK: stress-activated protein kinase; ERK: extracellular signal-regulated kinase; NF-κB: Nuclear factor kappa B; IL: interleukin; TNF-α: tumor necrosis factor; PBMCs: Peripheral Blood Mononuclear Cells; RT: reverse transcriptase; RNase H: Ribonuclease H; IN: integrase; NP: nucleoprotein; mir-146a: microRNA-146a; EC50: effective concentration 50%; TRAF6: TNF receptor-associated factor 6; IFN: Interferon; COX-2: Cyclooxygenase-2; NS1: nonstruc-tural protein 1; AMPK: 5′ adenosine monophosphate-activated protein kinase; IRF-3: Interferon regulatory factor 3; PARP: Poly (ADP-ribose) polymerase; SARS-CoV: Severe acute respiratory syndrome coronavirus; PLpro: papain-like protease; 3CLpro: 3-chymotripsin-like protease; SPR: Surface plasmon resonance; IC90: inhibitory concentration 90%; EBOV: Ebola virus; ROS: Reactive oxygen species; HS: heparan sulphate; HA: hemagglutinin; NA: neuraminidase; HIV: human immunodeficiency virus; CD: cluster of differentiation; NKT: natural killer T cells; CPE: cytopathic effect; IC50: inhibitory concentration 50%; CC50: cytotoxic concentration 50%; EC50: effective concentration 50%; EGCG: Epigallocatechin gallate; ECG: Epicatechin gallate; C5G: Catechin-5-gallate; PI3K: phosphoinositide 3-kinase; PDLKI1: pyruvate dehydrogenase lipoamid kinase isozyme 1; GSK3 β:glycogen synthase kinase-3β; LC3B:light chain 3-B; ROS: reactive oxygen species; MAP1: microtubule associated protein1; SI: selective index; G1,2: group 1,2; orf3a: open-reading-frame 3a; RT: reverse transcriptase; PBMC: peripheral blood mononuclear cells; IFN:interferon; NS1: non-structural protein 1; TF: theaflavin; TF-3-G:theaflavin-3-gallate; TF-3′-G:theaflavin-3′-G; TF-3,3′-DG: theaflavin-3,3′-DG; 3-DSC: 3-deoxysappanchalcone; GCG: gallocatechin gallate; Q3R: quercetin 3-rhamnoside; HMB: 2-hydroxy-3-methyl-3-butenyl alkyl; RANTES: regulated activation; normal T cell expressed and secreted; CG: catechin gallate; SEVI: semen-derived enhancer of virus infection; Env: envelope protein; gp: glycoprotein; TF2B: 3-isotheaflavin-3-gallate; TF3: theaflavin-3,3′-digallate; TPC: endo-lysosomal Two-Pore Channels.

## Data Availability

Not applicable.
